# Does self monitoring of blood glucose as opposed to urinalysis provide additional benefit in patients newly diagnosed with type 2 diabetes receiving structured education? The DESMOND SMBG randomised controlled trial protocol

**DOI:** 10.1186/1471-2296-13-18

**Published:** 2012-03-14

**Authors:** Helen M Dallosso, Helen C Eborall, Heather Daly, Lorraine Martin-Stacey, Jane Speight, Kathryn Realf, Marian E Carey, Michael J Campbell, Simon Dixon, Kamlesh Khunti, Melanie J Davies, Simon Heller

**Affiliations:** 1 Diabetes Research Department, University Hospitals of Leicester NHS Trust, Leicester, UK; 2 Department of Health Sciences, University of Leicester, Leicester, UK; 3 AHP Research, Hornchurch, UK/The Australian Centre for Behavioural Research in Diabetes, Melbourne, Australia/Centre for Mental Health and Wellbeing Research, Deakin University, Burwood, Australia; 4 School of Health and Related Research, University of Sheffield, Sheffield, UK; 5 Health Economics and Decision Science, School of Health and Related Research, University of Sheffield, Sheffield, UK; 6 Department of Cardiovascular Sciences, University of Leicester, Leicester, UK; 7 Department of Human Metabolism, University of Sheffield Medical School, Sheffield, UK

## Abstract

**Background:**

The benefit of self-monitoring of blood glucose (SMBG) in people with type 2 diabetes on diet or oral agents other than sulphonylureas remains uncertain. Trials of interventions incorporating education about self-monitoring of blood glucose have reported mixed results. A recent systematic review concluded that SMBG was not cost-effective. However, what was unclear was whether a cheaper method of self-monitoring (such as urine glucose monitoring) could produce comparable benefit and patient acceptability for less cost.

**Methods/Design:**

The DESMOND SMBG trial is comparing two monitoring strategies (blood glucose monitoring and urine testing) over 18 months when incorporated into a comprehensive self-management structured education programme. It is a multi-site cluster randomised controlled trial, conducted across 8 sites (7 primary care trusts) in England, UK involving individuals with newly diagnosed Type 2 diabetes.

The trial has 80% power to demonstrate equivalence in mean HbA1c (the primary end-point) at 18 months of within ± 0.5% assuming 20% drop out and 20% non-consent. Secondary end-points include blood pressure, lipids, body weight and psychosocial measures as well as a qualitative sub-study.

Practices were randomised to one of two arms: participants attend a DESMOND programme incorporating a module on self-monitoring of either urine or blood glucose. The programme is delivered by accredited educators who received specific training about equipoise. Biomedical data are collected and psychosocial scales completed at baseline, and 6, 12, and 18 months post programme. Qualitative research with participants and educators will explore views and experiences of the trial and preferences for methods of monitoring.

**Discussion:**

The DESMOND SMBG trial is designed to provide evidence to inform the debate about the value of self-monitoring of blood glucose in people with newly diagnosed type 2 diabetes. Strengths include a setting in primary care, a cluster design, a health economic analysis, a comparison of different methods of monitoring while controlling for other components of training within the context of a quality assured structured education programme and a qualitative sub-study.

**Trial registration:**

ISRCTN: ISRCTN95696668.

## Background

The prevalence of diabetes is increasing and it is estimated around 7.8% of the worldwide adult population will be affected by 2030 (up to 95% of whom will have type 2 diabetes (T2DM)) [1]. This growing burden on individuals, their families and healthcare systems emphasises the importance of developing effective, cost-effective and acceptable methods to manage this condition. Tight glycaemic control in people with diabetes can reduce a wide range of tissue complications including microvascular and large vessel disease [2]. Developing interventions that help people to self-manage their condition is vital. One component of diabetes self-management, self monitoring of blood glucose (SMBG) is considered by many to be essential but its precise role in the management of T2DM continues to evoke considerable controversy.

SMBG, hailed as one of the most important developments in diabetes care since the discovery of insulin, costs the UK National Health Service millions of pounds per year and the cost is rising [3]. For many with insulin treated diabetes and their families, the ability to measure blood glucose immediately is one of the essential management tools, enabling them to confirm suspected hypoglycaemic episodes or high glucose values rapidly and to take corrective action. However, over 30 years after its introduction, the degree to which SMBG contributes to improved metabolic control in non-insulin treated or newly diagnosed T2DM remains equivocal [4,5].

Structured education, which teaches the skills necessary to use information from SMBG to adjust insulin dose, can lead to sustained improvements in glycaemic control in individuals with Type 1 diabetes (T1DM), [6] although the contribution of SMBG within a complex intervention is unclear. Significant numbers of people with T1DM use SMBG to reflect on blood glucose levels, adjust their insulin dose and maintain optimum glycaemic levels. However, our clinical impression is that many individuals with T1DM religiously record the results but do nothing with them. Nevertheless, both patients and professionals would agree that SMBG technology should be available to everyone who uses insulin if only to detect and treat hypoglycaemia [7,8].

The situation is much more uncertain for individuals with T2DM, particularly those managing their condition with lifestyle adjustments or using oral agents other than sulphonylureas. This uncertainty is reflected in the wide variation in the use of SMBG in different parts of the UK, [9] but even in areas where SMBG is used less often, the estimated costs are considerable, amounting to hundreds of thousands of pounds in an average primary care trust. It has been argued that providing such technology to those on tablets or diet is a waste of time and money since there is little an individual can do with the result [10]. Others believe strongly that the information provided by SMBG is a powerful motivating factor, [11] encouraging self-management by enabling people with T2DM to measure directly the effect of different actions, such as the effect of eating on post-prandial glucose or the glucose lowering effect of exercise. Previous studies have attempted to assess the contribution of SMBG to improved metabolic control in this group. Most, [12,13] but not all, [14] observational studies have shown that even in those managing their T2DM using diet alone, increased frequency of SMBG is associated with better outcomes including lowered glycated haemoglobin (HbA1c) and less mortality. This has been used to advocate SMBG for all those with T2DM [15]. However, these results may merely indicate that those who are highly motivated (reflected in the take-up of SMBG technology) are likely to do well in the long term.

Trials of interventions incorporating education about SMBG have reported mixed results. The DIGEM study reported no evidence of an effect of SMBG (with or without support from trained practice nurses in the interpretation and application of results) [16]. However, limitations included potential contamination due to lack of cluster-randomisation, a drop in adherence to SMBG over the study period, [17] and exclusion of people with prior experience of SMBG. Similarly, the ESMON study reported no evidence of an effect of structured education with or without additional education on self-monitoring [18]. Both studies reported a net adverse effect on anxiety/depression, suggesting that SMBG increased worry and depressed mood.

Uncertainty in this field prompted NHS Diabetes to commission a multi-disciplinary working group to undertake a systematic review and meta-analysis of relevant trials and studies [4,5]. This reported a reduction in HbA1c of 0.21% from "simple" SMBG over no SMBG; a reduction of 0.52% from "enhanced" SMBG (with education, feedback, etc.) over no SMBG; and no difference between SMBG and urine testing [5]. In regard to cost-effectiveness, most studies did not allow for the potentially negative impact of SMBG on patient reported outcomes [5]. Those studies funded by manufacturers tended to be more favourable [5]. The estimated cost of SMBG in England is around £30 million a year. Studies that applied the most robust analyses, taking into account all costs, gains and disutilities, concluded that SMBG was not cost-effective [5]. However, what was unclear was whether a cheaper method of self-monitoring (such as urine glucose monitoring) could produce comparable benefit and patient acceptability for less cost.

Patient acceptability of the means of monitoring may be particularly important in sustaining effective self-management, thus the review included qualitative studies that had addressed this [5]. While SMBG was shown to have the potential to motivate and improve T2DM self-management in some people, it could also cause adverse psychological effects including depression and self-chastisement [5]. A failure to educate patients in interpretation and application of self-monitoring results was a consistent finding, along with a reported lack of interest in patients' self monitoring results from healthcare professionals (HCPs) and a frequent failure to act on the results [5]. People with newly diagnosed T2DM generally preferred blood to urine monitoring, finding it more hygienic and accurate though it should be noted that in the study quoted participants had not received structured education or consistent advice on what do to with the results [19]. Acceptability of method is likely to be influenced by perceptions of disease severity; necessity for self-management; accuracy and effectiveness of monitoring method; usefulness of results; convenience and disruption to daily life. When an educational element is involved, perceptions and experiences will be influenced by additional factors, [20] particularly the attitude of the HCPs delivering the education as well as those of the primary care team.

The review did not include one particular structured approach to monitoring, the Structured Testing Program (STeP) cluster-randomised trial (published recently) [21]. STeP reported a significantly greater reduction in mean HbA1c levels in people with T2DM allocated to 'structured' SMBG (using a tool to collect 7-point glucose profiles over 3 days) compared with those allocated to enhanced usual care including SMBG (-0.3% difference, and -0.5% difference per protocol analysis) [21]. Unlike DIGEM and ESMON, the STeP study demonstrated a significant reduction in depressed mood among those using the 'structured' SMBG in those with clinically significant depressed mood at baseline [21]. None of the interventions in the studies cited have involved *group *education and nor were they embedded in routine provision of education to those with newly diagnosed T2DM. Furthermore reports of these studies provide little information about the education involved in their intervention, and the training of educators.

In the UK the National Institute for Health and Clinical Excellence and the Diabetes National Service Framework now recommend that people with T2DM have access to structured education, [22,23] with an expectation that primary care trusts (PCTs) provide high quality courses for those newly diagnosed. It is important to establish the most appropriate and cost-effective form of self-monitoring for this group of patients. The aim of the DESMOND (Diabetes Education and Self Management for ongoing and Newly Diagnosed) SMBG Trial is to do this while controlling for the type and degree of education in self-management.

### Study objectives and outcome measures

The primary objective is to ascertain whether there are equivalent changes in HbA1c (primary outcome measure) in people with newly diagnosed T2DM allocated to either a urine or a blood glucose monitoring strategy over 18 months when incorporated as an integral part of a comprehensive self-management structured education programme (DESMOND).

The secondary objectives include: comparison of the two strategies on the secondary outcome measures over 18 months, including biomedical measures (systolic and diastolic blood pressure, lipid profile, body weight and waist circumference) and psychological measures (well being, anxiety, depression, treatment satisfaction and perceptions about diabetes) (for further details see Table 1). A qualitative sub-study will explore participant views about acceptability of, and preference for, the two self-monitoring methods included. An assessment of cost effectiveness will be conducted.

**Table 1:** Biomedical and questionnaire measures used and time points in the trial

	Baseline	6 months	12 months	18 months
**Biomedical measures**				
HbA1c (mmol/mol)	✓	✓	✓	✓
Systolic blood pressure (mmHg)	✓	✓	✓	✓
Diastolic blood pressure (mmHg)	✓	✓	✓	✓
Total cholesterol (mmol/l)	✓			✓
HDL cholesterol (mmol/l)	✓			✓
LDL cholesterol (mmol/l)	✓			✓
Triglycerides (mmol/l)	✓			✓
Weight (kg)	✓	✓	✓	✓
Waist circumference (cm)	✓	✓	✓	✓
**Questionnaire measures**				
The Well-being Questionnaire 28 (W-BQ28) [24,25]	✓	✓	✓	✓
Brief Illness Perceptions Questionnaire (BIPQ) - diabetes version [26]	✓	✓	✓	✓
Short-Form 36 (SF-36) [27]	✓			✓
Summary of Diabetes Self-Care Activities (SDSCA). [28]		✓	✓	✓
Euro-Qol (EQ-5D) [29]	✓	✓	✓	✓
Confidence in Diabetes Self-care (CIDS) [30]		✓	✓	✓
Diabetes Treatment Satisfaction questionnaire (DTSQ) [31]	✓	✓	✓	✓
Beliefs about self monitoring*		✓	✓	✓

*Set of questions on beliefs and attitudes towards self monitoring. Questions were developed for the DiGEM study and have been adapted by Dr Jane Speight with kind permission from Dr Andrew Farmer

### Presentation of the hypothesis

Our null hypothesis is that use of SMBG rather than urine monitoring in the context of delivering the DESMOND intervention will not be equivalent in terms of improving and sustaining glycaemic control over 18 months.

### Testing the hypothesis

We will be measuring the equivalence of SMBG (and its cost-effectiveness) over a clinically relevant period of follow-up while controlling for the other components of a complex intervention, in particular, the educational programme itself and feedback of glucose levels from another form of monitoring.

### Implications of the hypothesis

The DESMOND SMBG trial will provide additional evidence to inform the debate around the value of SMBG for people with T2DM not using insulin or at risk of hypoglycaemia. In particular it will allow us to assess whether SMBG is equivalent to urine monitoring in people with newly diagnosed T2DM who are undertaking a quality assured structured education course delivered in primary care.

If the null hypothesis is not rejected, then SMBG will not be recommended for people with newly diagnosed T2DM. However, if the null hypothesis is rejected, and the results demonstrate that SMBG is equivalent to urine monitoring in terms improving and sustaining glycaemic control over 18 months, then this will be used as evidence to recommend SMBG for people with newly diagnosed T2DM who are undertaking the DESMOND intervention.

## Methods/Design

The study is an 18 month multi-site cluster randomised controlled trial conducted in 8 sites in 7 PCTs in England, and managed by a central DESMOND Research Office. Ethical approval was received from Cambridgeshire Research Ethics Committee (07/H0304/129) and local research governance approval from participating PCTs.

Practices are randomised to one of two arms: 1) Participants attend a DESMOND structured education programme which includes a module on self-monitoring of blood glucose; and 2) Participants attend a DESMOND structured education programme which includes a module on self-monitoring of urine glucose.

### Setting and recruitment of PCTs

The study is being conducted in primary care in a cross-section of settings ranging from inner city to rural PCTs. Two criteria were used when approaching PCTs about participating in the study. First, they were required to have a team of accredited DESMOND educators who were interested and willing to participate. Second, PCTs needed to be willing to support the prescription requirements of the study. PCTs vary in their policy on the prescription of blood glucose monitoring strips for people with non-insulin treated T2DM, which can be a contentious issue due to the prescribing costs involved. Practices are refunded the cost of prescriptions for test strips to prevent the cost being a barrier to self-monitoring within the trial.

### Randomisation of practices

The study is cluster-randomised, with all enrolled participants in one practice randomised to one arm of the trial in order to avoid potential 'contamination' from participants randomised to different arms. Randomisation was carried out independently at the University of Leicester, after stratification for practice setting and list size. Two randomisation plans were computed, one for small and one for large lists sizes. ^a^Practices agreed to participate in the study before being randomised. Practices were not informed of their randomisation when recruited, but become aware of it during the study as participants attend the practice for clinical support and repeat prescriptions for monitoring resources.

### Patient eligibility criteria

People with newly diagnosed T2DM were referred by their practice within 12 weeks of diagnosis. They were excluded if they were under 18 years, were taking insulin, had severe and enduring mental health problems, were not primarily responsible for their own care, were unable or unwilling to participate in a group programme (for example, housebound or unable to communicate in English), or were participating in another research study. They were not eligible if they had already started SMBG, (unless this involved informal 'trial and error' without instruction from a HCP and they were willing to be randomised to either type of monitoring). Participants were required to attend a DESMOND programme within 6 months of diagnosis.

### Referral and recruitment of participants

Practice nurses referred people who were newly diagnosed with T2DM and interested in participating in the study to the local DESMOND team. A member of the local DESMOND team then contacted each individual referral to confirm eligibility and check readiness to participate; if willing the individual was booked to attend an appropriate DESMOND programme. Practice nurses and DESMOND co-ordinators were instructed not to tell individuals which type of course they would be attending.

### Collection of baseline and follow-up data

Participants were sent a questionnaire booklet containing a set of questionnaire tools (Table 1) which they were asked to complete and bring to the programme. Upon attendance, prior to the programme commencing, written informed consent was taken by the educators. Participants were informed which arm of the study they were allocated to after informed consent was established and the programme started.

Biomedical data collected at diagnosis by the primary care team (see Table 1) were sent to the local DESMOND team. These constituted baseline data for participants who consented to join the study.

Follow-up data are collected by practice nurses at 6, 12 and 18 months post-programme. Two weeks before each follow-up date, the DESMOND Research Office sends participants a letter asking them to complete and return an enclosed questionnaire. The letter also asks them to contact their general practice and arrange an appointment for the study measures to be made. The practice is sent a letter at the same time, asking them to collect the information and return it to the research office. A reminder letter and questionnaire are sent to the participant after four weeks if the questionnaire has not been returned; practices who do not return follow-up data are contacted and prompted for the information. If, for some reason, data are not collected at a follow-up point, the participant is approached at the subsequent follow-up, unless they have been formally withdrawn from the trial.

### Study intervention

The study intervention is the DESMOND Newly Diagnosed structured education programme with specific sessions on SMBG, compared with the same programme with specific sessions on self-monitoring of urine glucose [32]. The standard programme involves 6 h of contact time, facilitated by two trained HCPs. The content of the curriculum is designed for people attending within 12 weeks of diagnosis and focuses on cardiovascular risk factors and associated lifestyle factors, such as food choices and physical activity. The broad content of the curriculum has been reported elsewhere [33]. The HCPs attend a formal training programme in order to be accredited as DESMOND Educators [34]. A quality development pathway and specifically developed and tested quality assurance tools are used to ensure consistency of delivery.

The standard DESMOND Newly Diagnosed programme includes a 20 min session on self-monitoring which covers the difference between short term and long term monitoring of blood glucose, without detailed coverage of the different methods, and forms a relatively small part of the programme. However, for the purpose of the trial, the session on self monitoring has been extended in length and content. The development, piloting and content of the self-monitoring sessions for the trial and the training of the DESMOND educators are described below.

### Development and piloting of self-monitoring sessions

Experienced DESMOND trainers developed and piloted the new self-monitoring sessions using an iterative cycle. Six educators from 3 PCTs were then trained in delivery of the sessions before delivering it incorporated into the DESMOND programme to patients in their area. Subsequently the educators provided feedback which led to revision of the sessions and resources.

The 6-h DESMOND structured education programme is underpinned by a philosophy and learning theories that promote adult learning through interactive step-wise processes, which increase self-efficacy to promote self-management [33]. Therefore, the newly developed monitoring sessions needed to reflect these principles.

The content of the self-monitoring sessions is summarised in Table 2. It was delivered in two separate sessions, totalling 100 min in length. They were embedded within the DESMOND education programme, which for the purpose of this study is delivered in two sessions, one to two weeks apart. Resources were developed for use within the sessions to enhance participant interaction and self confidence, support a reflective approach to self-monitoring, and identify self management changes in relation to results. The approach to self-monitoring is not directive and regimented but promotes the practice of self-monitoring as a tool to support decision making in regard to lifestyle changes and medication. Participants were encouraged to decide when and how often to monitor, how to interpret the results and to explore options for change. Although they were allocated randomly to either the blood or urine method of monitoring, they are able to swap methods or to stop monitoring if they choose. Participants were asked to report any change in method on the 6, 12 or 18 month questionnaire.

**Table 2:** Contents of sessions on self-monitoring included in DESMOND programme in trial

Session 1	Monitoring and measuring blood glucose levels (20 mins)
	
	• Long term blood glucose control using HbA1c and recommended targets
	• How measurement of long term glucose control differs from self monitoring
	• Introduction to Health Profile
	
	Methods of self monitoring (20 mins) *
	
	• Purpose of self monitoring and how to use the information
	• How and when to monitor blood/urine
	• Target levels for blood/urine monitoring
	
	Purpose of self monitoring (20 mins)
	
	• Purpose of self monitoring and how each individual can use the information he/she gathers
**Session 2 (1 to 2 weeks later)**	Monitoring, feedback and action planning (40 mins)
	• How to interpret test results, identify personal targets and act on them
	• Key messages related to self-monitoring
	• Importance of confidence score
	• How to action plan for self monitoring

* Content varied depending on whether the session was covering urine or blood monitoring

### Methods of self-monitoring

The first session involves experiential learning with the method of self-monitoring allocated. Participants in the blood monitoring arm were taught how to use the Ascensia Contour meter; those in the urine monitoring arm were taught how to use Diastix urine strips and all participants were provided with two pots of test strips. In the second session they were encouraged to discuss their monitoring experiences and challenges. During the study participants obtain further supplies of strips by repeat prescription from their practice. The majority of study participants are exempt from prescription charges, but there is a procedure in place to enable those who are not exempt to reclaim the cost.

### Training of educators

To maximise fidelity of the intervention, educators were trained to deliver these expanded monitoring sessions. The training consisted of an initial training course (1 1/2 days) which included modelling of delivery of the self-monitoring sessions by the trainers. A second training course (1 day) halfway through the study provided an update and the opportunity to deliver the sessions to their peers and receive feedback.

The training included a session designed specifically to: 1) inform educators about the concept of equipoise - agreement in the scientific community that there is genuine uncertainty whether one treatment is more beneficial than another, [35] and 2) raise educators' awareness of their own views about the two monitoring methods (i.e. the two arms of the trial). This included reflection on how their personal beliefs, attitudes and behaviours might impact on their delivery of the sessions and in turn the outcome of the study. It is important to note that the training was not designed to achieve a position where every educator was considered to be in individual equipoise regarding self-monitoring. Rather, it enabled educators to explore and recognise their personal beliefs and how these beliefs may impact on their delivery of the intervention, and to learn behaviours that would prevent this type of impact.

The educators' beliefs about self-monitoring were assessed using a bidirectional linear scale [36]. The scale consisted of a 5 m ribbon placed on the floor with the ends labelled 'blood monitoring' and 'urine monitoring' and the centre labelled 'neutral'. The educators were asked to position themselves on the line in a place that reflected their views and beliefs about the two methods of monitoring. Initially the task was done individually without other educators observing; the task was then used in a group setting to create an opportunity for discussion of the concept of equipoise and the potential impact of beliefs on delivering the intervention.

During the trial, DESMOND trainers observed the educators delivering the sessions in order to ensure that delivery reflected the curriculum and was not biased with regards to the method of monitoring, and to provide feedback and support to the educators.

### Involvement of practice nurses

Practice nurses based in participating practices are involved in providing care and support for participants over the 18 months of the study. They also provide repeat prescriptions for monitoring strips. A total of 75 practices are taking part in the study and it was therefore not possible to provide extensive training to the practice nurses involved. Each nurse received a one hour visit from a member of the research team which covered the study's standard operating procedures for the collection of data and the importance of being impartial and neutral when they discussed monitoring methods with the participant. They were encouraged to follow the approach used by DESMOND educators to help the participant self-manage their diabetes.

### Analysis

#### Power calculations and sample size

The aim is to show equivalence in mean HbA1c at 18 months of within ± 0.5%. Assuming a standard deviation (SD) at 18 months of 1.5%, [37] the sample size required without allowing for clustering for 80% power at 5% significance is 142 per arm. With an intra-cluster correlation of 0.05, [38] and an expected mean cluster size of 5, adjustment for clustering brings the number required to 170 per arm. Assuming 20% drop out and 20% non-consent, the numbers required are 266 referrals and 213 consents per arm.

#### Statistical analysis

There will be double entry of all data and discrepant values will be investigated. Results will be reported according to CONSORT guidelines for cluster randomised trials and statistical analysis carried out on an intention-to-treat basis. Missing outcomes will not be replaced and an average over-time of continuous outcomes will be derived. This procedure will measure the cumulative effect of the treatment and have the maximum number of participants. Continuous variables will be analysed using a linear model adjusting for clustering using generalised estimating equations, and adjusting for baseline values.

#### Economic evaluation

The evaluation will be undertaken from the NHS perspective. The health service use costs of the two strategies will be calculated at 18 months in both groups. Costs incurred between 12 and 18 months will be discounted at 3.5%, in line with NICE guidelines. The mean cost of the two groups over 18 months will be compared using non-parametric tests. Results will be reported with 95% confidence intervals and uncertainty will be examined using sensitivity analysis.

An incremental cost-effectiveness ratio will not be calculated over the 18 months of the intervention as this length of follow-up does not capture all relevant costs and outcomes. Consequently, without the use of modelling, a cost-effectiveness analysis could be misleading. The need or otherwise for cost-effectiveness modelling will depend on there being economically important clinically significant differences in glycaemic control, as determined by the likely impact on and hence, long-term effects on morbidity and mortality. Whether any differences in glycaemic control are important will be informed by reviewing the cost-effectiveness of other interventions that have been evaluated using lifetime models. If such differences do exist, the modelling of the longer-term effects to assess the incremental cost per quality adjusted life-year will be conducted.

#### Qualitative sub-studies

In order to investigate the acceptability of the two monitoring methods, qualitative semi-structured interviews are being conducted with a small sample of participants including a similar number of those allocated to each monitoring method in the trial. The focus of this is to explore participants' views and experiences of using the allocated monitoring method (and the other method if they swapped) as part of their self-management of T2DM. Interviews are conducted approximately one year after attending the DESMOND SMBG programme.

In addition, focus groups and qualitative interviews are being conducted with a sample of the DESMOND educators involved in delivering the DESMOND SMBG intervention at different points throughout the trial period. The purpose of this is to explore educators' views and experiences of the trial including: the training and their delivery of the intervention, their views and preferences with regards to the two monitoring methods, and their reflections on delivering both arms of a trial while potentially not being in individual equipoise.

## Discussion

The DESMOND SMBG trial has been designed to provide additional evidence to inform the debate around the value of SMBG for people with Type 2 diabetes (T2DM) not using insulin or at risk of hypoglycaemia. Its particular strength is that it will allow us to assess the additional contribution of SMBG in people with newly diagnosed T2DM who are undertaking a quality assured structured education course delivered in primary care. Our hypothesis is that use of SMBG rather than urine monitoring in the context of delivering the DESMOND intervention will provide no added benefit in improving and sustaining glycaemic control over 18 months. Thus, we will be measuring any added benefit of SMBG (and its cost-effectiveness) over a clinically relevant period of follow-up while controlling for the other components of a complex intervention, in particular, the educational programme itself and feedback of glucose levels from another form of monitoring.

We will also compare the effect of the two self-monitoring methods on secondary outcome measures, including biomedical (systolic and diastolic blood pressure, lipid profile, body weight and waist circumference), and psychological (generic and disease specific well being, treatment satisfaction and perceptions about diabetes) measures. We will explore the views of participants concerning their experience of and preference for the two methods, and measure which self-monitoring strategy participants adopt over the subsequent 18 months. We will calculate the cost effectiveness of both methods.

Attitudes to self-monitoring are influenced by people's experience of the real world, as is the success or otherwise of their self-monitoring strategies. While our study is carefully planned and monitored, by virtue of the fact that it is embedded in the real world, it is in essence a pragmatic trial. The incorporation of questionnaires investigating psychosocial aspects and impact of self-monitoring will provide an important contribution to questions about acceptability of the methods; the findings of which will help illuminate the results of the main trial. The questionnaires will both record and monitor changes in self-monitoring strategies among the participants and the qualitative sub-study will seek a greater understanding of participant attitudes and preferences in respect of self-monitoring. Since the DESMOND education programme has been shown to be active in changing illness beliefs, [32] we will also be examining the benefit of SMBG within an education programme which has been set up to enable participants to exploit the benefits of feedback from direct measurement of their glucose levels. Finally to prevent potential contamination from attitudes of practice clinical staff we have undertaken cluster randomisation at the level of the practice.

Our trial is also exploring the effect of specific attention to the potential influence of educators' attitudes upon intervention delivery through targeted training and through assessment of attitudes and preferences. The training highlighted issues of potential bias in intervention delivery by specifically raising awareness of equipoise and by formally assessing educators' delivery of the intervention in both arms of the trial. A qualitative sub-study with the educators will enable us to investigate their attitudes towards and experiences of both the training they received and delivery of the intervention(s).

## Endnote

^a^Randomisation was computed using the online resource at http://www.tufts.edu/~gdallal/randomize.htm.

## Abbreviations

SMBG: Self monitoring of blood glucose; T1DM: Type 1 diabetes; T2DM: Type 2 diabetes; HbA1c: Glycated haemoglobin; PCT: Primary care trust; DESMOND: Diabetes education and self management for ongoing and newly diagnosed; HCP: Healthcare professional.

## Competing interests

HMD, HCE, HD, LMS, KR, MEC, MJC, and SD declare that there are no competing interests.

JS is a member of the Australian Accu-Chek Advisory Board (Roche Diagnostics) and has previously been the recipient of an unrestricted educational grant, funding for conference travel and consultancy fees. Her research centre has also received sponsorship from Roche Diagnostics and Abbott Diabetes Care toward the costs of hosting the 2011 Symposium for Behavioural Research in Diabetes (Perth, Sept 2011).

MJD has acted as consultant, advisory board member and speaker for Novartis, Novo Nordisk, Sanofi-Aventis, Lilly, Merck Sharp & Dohme and Roche, and as a speaker for Servier. She has received grants in support of investigator and investigator initiated trials from Novartis, Novo Nordisk, Sanofi-Aventis, Lilly, Pfizer, Merck Sharp & Dohme, GlaxoSmithKline and Servier.

KK has acted as a consultant and speaker for Novartis, Novo Nordisk, Sanofi-Aventis, Lilly and Merck Sharp & Dohme. He has received grants in support of investigator and investigator initiated trials from Novartis, Novo Nordisk, Sanofi-Aventis, Lilly, Pfizer, Boehringer Ingelheim and Merck Sharp & Dohme.

SRH has served on advisory boards and given talks at meetings on behalf of Abbot and Lifescan for which his institution has received fees. He also chaired the NHS Diabetes working group which reported on SMBG in Type 2 diabetes in 2009.

## Authors' contributions

All authors (except HCE) participated in the design of the study protocol. HMD and KR managed the study and co-ordinated the collection and processing of data. HD and LMS designed the study intervention and trained the Educators. HCE, HMD, HD, LMS and SRH drafted the manuscript and all authors read, edited and approved the final document.

## Pre-publication history

The pre-publication history for this paper can be accessed here:

http://www.biomedcentral.com/1471-2296/13/18/prepub
